# Expression of natriuretic peptide receptor-A in esophageal squamous cell carcinomas and the relationship with tumor invasion and migration

**DOI:** 10.1186/1477-7819-12-154

**Published:** 2014-05-21

**Authors:** Zhilong Zhao, Haoqian Liu, Ya Yang, Kai Sun, Min Li, Jia Zhang, Hui Cai, Jiansheng Wang

**Affiliations:** 1 Department of second Thoracic surgery, First Affiliated Hospital, Xi'an Jiaotong University, Yanta West Road no. 277, Xi'an, Shaanxi 710061, China; 2 Department of Surgical Oncology, Baoji Central hospital, Jiang Tan Road no. 8, Baoji, Shaanxi 721000, China

**Keywords:** natriuretic peptide receptor-A, NPRA, esophageal squamous cell carcinoma, ESCC, MMP, invasion, migration

## Abstract

**Background:**

The natriuretic peptide receptor-A (NPRA) has been investigated as a receptor of natriuretic peptides in the cardiovascular system. In this study, however, we analyze the expression status of NPRA and the relationship with tumor invasion in esophageal squamous cell carcinoma (ESCC) for the first time.

**Methods:**

Western blots were used to examine the expression status of protein in human ESCC cell lines. Then, we used immunohistochemistry to detect the expression of NPRA in 45 ESCC specimens and 40 corresponding nontumor tissues. The clinical data were analyzed through statistical methods. Sh-RNA-NPRA was transfected into Eca109 cells to detect the relationship between NPRA and cell invasion through transwell assays.

**Results:**

In esophageal squamous cells, the expression of NPRA was strongly detected in the cytoplasm, while undetectable or very weak in the nucleus. The positive rates of NPRA in cancer tissues are significantly higher than that in nontumor tissues (*P* <0.05). Clinicopathological analyses revealed that increased NPRA expression correlated with differentiation and TNM stage (*P* <0.05), while it showed no statistically significant association with age, gender, and lymph node metastasis. In analysis of prognosis, we found that highly.Transwell assays showed that NPRA promoted Eca109 cell migration and invasion *in vitro* and may be involved in MMP2 and MMP9 activation.

**Conclusions:**

NPRA protein is highly expressed in ESCC tissues and could promote Eca109 cell migration and invasion *in vitro*.

## Background

Esophageal cancer is one of the most prevalent cancers in the world; at the same time, it is one of the most aggressive digestive tract tumors and ranks sixth in cancer-related deaths. As the most frequent histological subtype of esophageal cancer, esophageal squamous cell carcinoma (ESCC) shows geographic variations in incidence, representing about 80% morbidity for the cases in Asia, especially in Shanxi and Henan in China [1]. Unfortunately, the prognosis of patients with ESCC is relatively poor, with the 5-year overall survival rate being below 15% [2,3]. Although, more and more antitumor therapies have been used in patients with ESCC, the prognosis still remains poor. Therefore, by exploiting a new cancer-targeting drug, we may improve the prognosis and life quality for ESCC patients. Recently, an increasing number of studies have discovered that there is a significant connection between heart hormone and malignancy [4-6].

The natriuretic peptide receptor-A (NPRA) is one of the receptors of natriuretic peptides [7]. It has been recognized for several decades due to its general effects on the cardiovascular system, such as natriuretic, diuretic, vasorelaxant, and anti-proliferative responses that alter intracellular levels of cGMP [8,9], which in turn, affects cell growth, proliferation, apoptosis, and inflammation through cGMP-regulated transcription factors, ion channels, phosphodiesterases, and possibly other effector proteins [10-12]. Until now, more and more studies have focused on the functional roles of NPRA in renal, cardiovascular, endocrine and skeletal homeostasis [13]. But recently, many in-depth research studies showed that NPRA may play a carcinogenic role in various solid tumors. For example, NPRA was found to be frequently expressed in human prostate cancer (PCa) cells, and downregulation of NPRA induced apoptosis in PCa cells. Furthermore, NPRA deficiency can substantially protect C57BL/6 mice from lung, skin, and ovarian cancers [14].

More and more studies have shown that matrix metalloproteinases (MMPs) may play an important role in the invasion and metastasis of a variety of malignant tumors [7]. MMP2 and MMP9 are two important members of the MMP family, and overexpression of MMP2 and MMP9 has been confirmed to associate with a variety of malignancies that contain esophageal squamous cell carcinomas [15].

Therefore, NPRA may play an important role in malignancy, but until now, the actual frequency of NPRA expression and cellular localization in ESCC remained unknown. So, in this study, we investigated the expression and localization of NPRA in tissues and cells from ESCC patients, and discuss the mechanism of NPRA in esophageal squamous cell carcinomas.

## Methods

### Cell lines

Human ESCC cell lines Eca109 and TE-1 were purchased from Shanghai Institute of Biochemistry and Cell Biology (Shanghai, China). One human immortalized normal esophageal epithelial cell line (Het-1A), which was used as a ‘normal’ control for ESCC cell lines, was maintained in our laboratory. Eca109 and TE-1 cells were cultured in RPMI1640 (Hyclone) supplemented with 10% fetal bovine serum (Hyclone), 100 U/ml penicillin and 100 μg/ml streptomycin, within a humidified atmosphere containing 5% CO_2_ at 37°C. Het-1A cells were cultured in bronchial epithelial basal medium with growth supplements (Clonetics).

### Patients and tissue samples

Samples were obtained from the first Affiliated Hospital of Xi'an Jiaotong University. The research was approved by the ethics governing board of our hospital. For studies involving humans have all signed information consent form. The patients who received preoperative chemotherapy and radiotherapy and who had a history of an associated malignant tumor were all excluded. All samples had been clinically and histopathologically diagnosed with ESCC by surgery and pathology. A total of 85 cases were selected that contained 45 ESCC specimens and 40 corresponding nontumor tissues from January 2004 to December 2006. The ESCC specimen group consisted of 35 males and 10 females, ranging in age from 49 to 72 years (mean 64 years). Fourteen cases were classified as highly differentiated squamous carcinoma, and 31 cases were categorized as moderately or poorly differentiated squamous carcinoma. All resected specimens were fixed in 10% formaldehyde solution and embedded in paraffin. Five micrometer sections were cut and mounted on glass slides.

### Immunohistochemical staining

NPRA was evaluated by immunohistochemical method according to the following instructions. The sections were baked at 60°C for 2 h, dewaxed in xylene, hydrated in gradient alcohol, and washed by 0.01 mol/l citrate buffer (pH 6.0). To enhance staining, slides were subjected to microwave antigen retrieval in 0.01 M sodium citrate buffer (pH 6.0) at 121°C for 20 min and the endogenous peroxidase was inhibited by 3% H_2_O_2_. The slides were incubated overnight at 4°C with anti-NPRA (1:200; SC-16867, Santa Cruz, California, USA). Normal goat serum was used as a negative control. After being washed in PBS, tissue sections were treated with the corresponding secondary antibody and visualized by incubation with 3,3-diaminobenzidine (DAB). Then, the sections were counterstained with hematoxylin and mounted.

### Immunohistochemical staining and scoring

All slices were evaluated by three pathologists without knowledge of the clinical outcome. The percentage of immunoreactive cells was graded on a scale of 0 to 4: 0 for ≤5% positive tumor cells; 1 for 6 to 25% positive tumor cells; 2 for 26 to 50% positive tumor cells; 3 for 50 to 75% positive tumor cells; and 4 for ≥76% positive tumor cells. The staining intensities were graded from 0 to 3: 0 for no staining; 1 for weak staining (light yellow); 2 for moderate staining (yellow-brown); and 3 for strong staining (brown). So, the final score came from multiplying the quantity and intensity scores. For final scores, 0 was considered negative, 1 to 3 was regarded as weakly positive and 4 to 8 was regarded as strongly positive.

### Western blot analysis

The expression of NPRA protein in human ESCC cell lines was detected by western blot. Total proteins were extracted from Eca109, TE-1 and Het-1A with Cell Lysis Buffer containing Protease Inhibitor Cocktail and PMSF. About fifty micrograms of protein was separated by sodium dodecyl sulfate polyacrylamide gel electrophoresis (SDS-PAGE) on a 10% polyacrylamide gel and transferred onto polyvinylidene fluoride (PVDF). Membranes were blocked for 90 min with a 5% milk solution prepared in PBS, followed by incubation overnight at 4°C with the primary NPRA antibodies (1:500, SC-16867, Santa Cruz, CA, USA) and β-actin antibodies (1:200, BS0061R, bioss, Beijing, China). These were then incubated with horseradish peroxidase-conjugated secondary antibodies and visualized by enhanced chemiluminescence (ECL) (Millipore, Massachusetts, Billerica, USA).

### Establishment of stable natriuretic peptide receptor-A knockdown cells

Eca-109 cells were transfected with control sh-RNA or sh-RNA-NPRA, which includes sh-RNA-NPRA-NC, sh-RNA-NPRA-21897, sh-RNA-NPRA-21898, and sh-RNA-21899. All sh-RNA was purchased from GeneChem company. Cell transfection was performed using Tfx-20 (Promega, Madison, WI, USA) according to the manufacturer protocol.

### Migration and invasion assay

Cell migration and invasion were tested in transwell chambers, which were coated without or with Matrigel (BD Biosciences) on the upper surface. Eca109 cells (5 × 10^4^ or 10 × 10^4^) that had been treated with the control medium for 24 h were plated into the upper chamber after transfection; serum was added to the bottom wells of the chambers to induce cell migration. After incubation for 8 h or 24 h, the cells that had migrated or invaded through the membrane to the lower surface were fixed by 10% formaldehyde solution, stained with 0.5% crystal violet hydrate solution and counted.

### Statistical analysis

All statistical analyses were performed using SPSS 18.0 software (IBM, Chicago,USA). The expression of NPRA and clinicopathological characteristics was evaluated by Chi-square test. Student’s *t*-test was used to evaluate measurement data. The accepted level of significance was *P* <0.05.

## Results

### Expression of natriuretic peptide receptor-A in human esophageal squamous cell carcinoma tissues and cells was apparently higher than in noncancer tissues and cells

**Figure 1:**
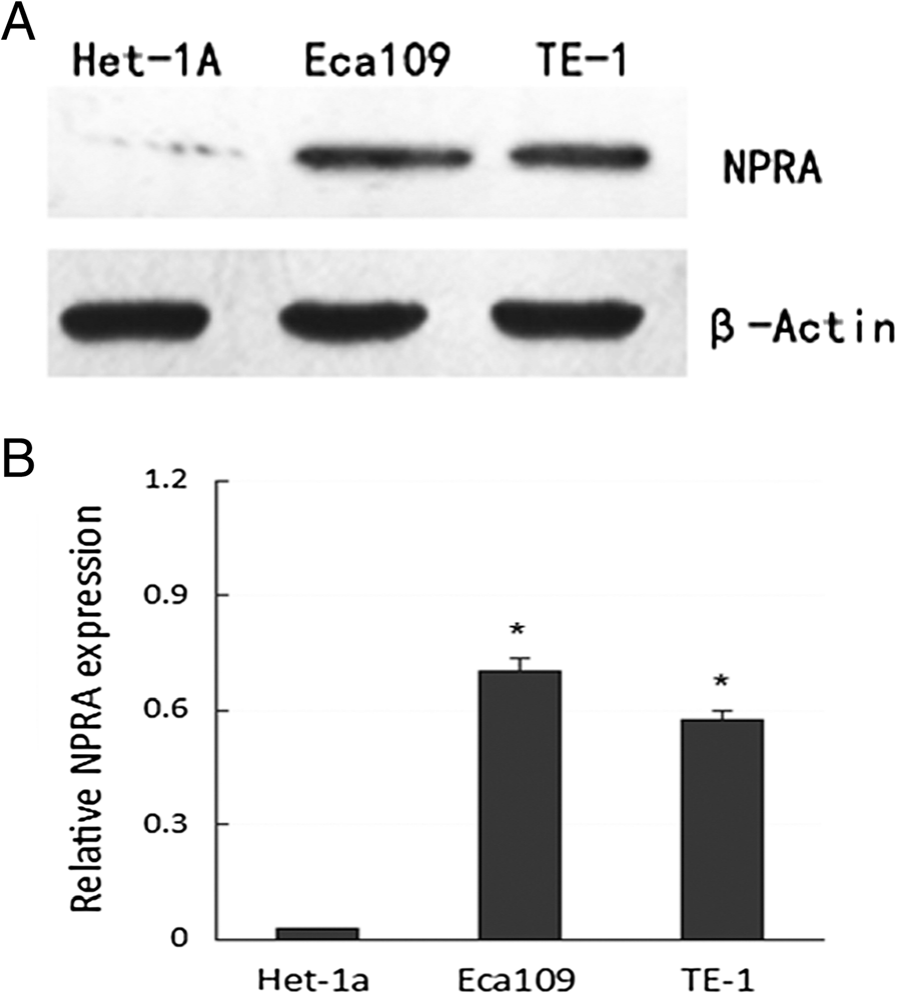
**Expression of natriuretic peptide receptor-A (NPRA) protein in esophageal squamous cell carcinoma (ESCC) cell lines (Eca109, TE-1) and normal esophageal epithelial cell line (Het-1A). (A)**: western blot figure, **(B)**: Bar chart from **A**. Data are presented as mean ± SD; *P <0.05 compared with normal epithelial cell (NC) group using the Student’s *t*-test.

Western blot was conducted to detect NPRA protein expression in two human ESCC cell lines and normal epithelial cells. We found that the two ESCC cell lines showed a significantly higher expression level of NPRA protein than human normal epithelial cells (*P* <0.05). Additionally, the expression of NPRA protein in Eca109 and TE-1 revealed no differences (Figure 1A, B).

**Figure 2:**
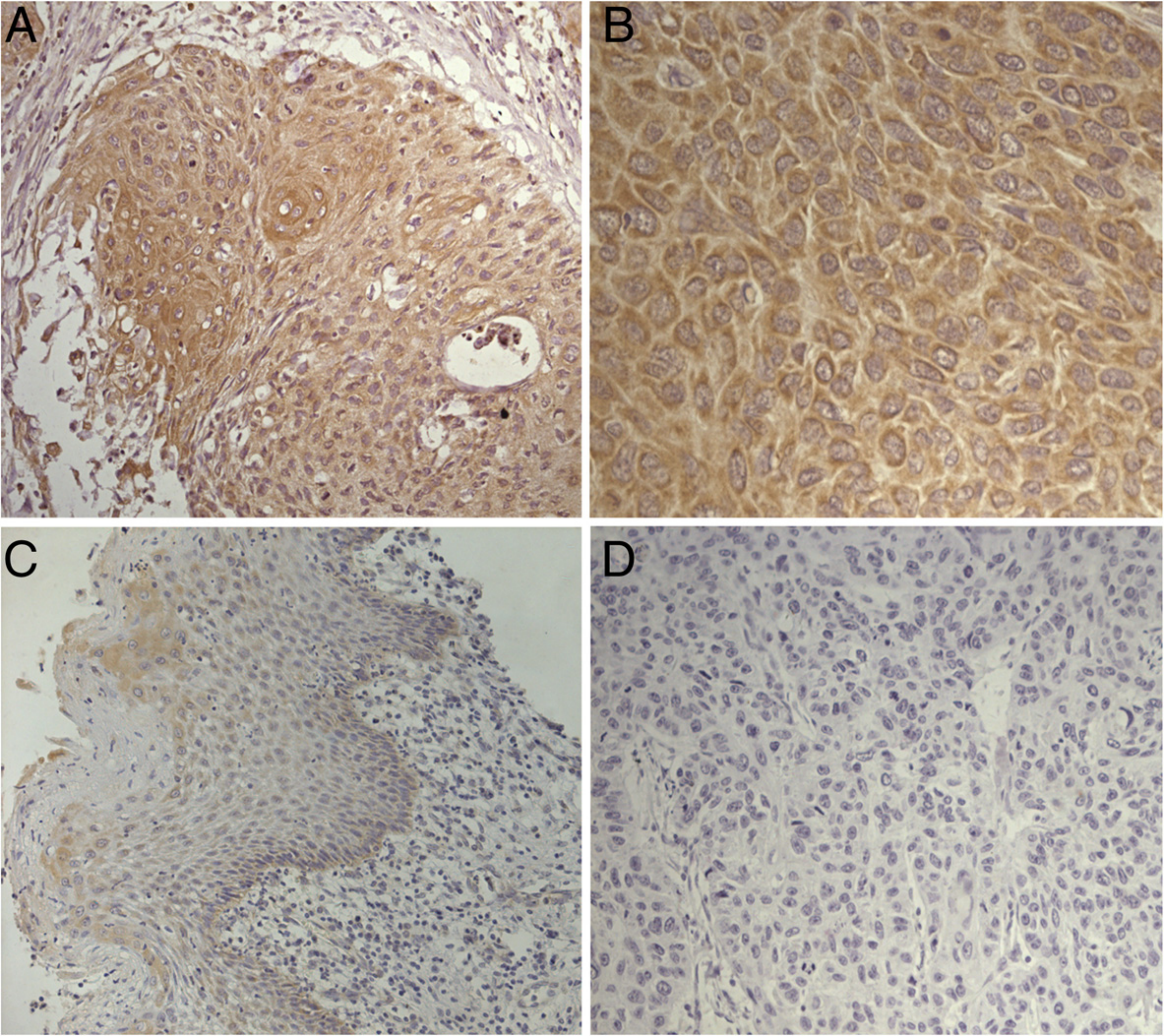
**Immunohistochemical staining for natriuretic peptide receptor-A (NPRA) in esophageal squamous cell carcinoma (ESCC) tissues and non-cancerous tissues. (A)** Strongly positive staining in a primary ESCC (magnification × 200), **(B)** Strongly positive staining in a primary ESCC (magnification × 400), **(C)** negative staining in non-cancerous tissues (magnification × 200), **(D)** negative staining in negative control group (magnification × 200).

Immunohistochemical results demonstrated that NPRA protein was highly expressed in 32(71.1%) of 45 human esophageal squamous tissues, with lower expression present in 7(17.5%) of 40 corresponding human nontumor tissues (Table 1). NPRA protein was mainly expressed in the cytoplasm and cytomembrane (Figure 2).

**Table 1:** The expression of natriuretic peptide receptor-A (NPRA) in human esophageal squamous and non-tumor tissues

**Samples**	**n**	**NPRA (n(%))**		***χ***^**2**^	***P***
		**High**	**Low**		
Squamous tissues	45	32(71.1)	13(28.9)	24.512	0.000^a^
Nontumor tissues	40	7(17.5)	33(82.5)		

^a^*P* <0.05 refelectd that there has statistical significance in two groups.

### The clinicopathological features of natriuretic peptide receptor-A expression in esophageal cancer

We also investigated the association between highly positive NPRA expression and clinicopathological factors of the tumor. The results revealed that higher positive expression of NPRA correlated with the TNM stage (*P* = 0.027) and histologic differentiation (*P* = 0.000). There was no significant association among NPRA protein expression and age, sex, lymph node metastases, or location (Table 2).

**Table 2:** Relationship of natriuretic peptide receptor-A (NPRA) expression with pathological parameters of cancer

**Clinical parameters**	**N**	**NPRA (%)**		***χ***^**2**^	***P *** **value**
		**High**	**Low**		
Age				0.007	0.933
<60	16	12	4		
≥60	29	20	9		
Gender				0.234	0.629
Male	35	26	9		
Female	10	6	4		
Lymphatic metastasis				0.265	0.267
Yes	20	15	5		
No	25	17	8		
Histologic differentiation				17.902	0.000^a^
G1	14	4(28.6)	10(71.4)		
G2 + G3	31	28(90.3)	3(9.7)		
TNM Stages				4.881	0.027^a^
I	15	7(46.7)	8(53.3)		
II + III	30	25(83.3)	5(16.7)		
Location				0.585	0.747
Proximal	10	7	3		
Middle	21	16	5		
Distal	14	9	5		

^a^*P* <0.05 refelectd that there has statistical significance in two groups.

### Natriuretic peptide receptor-A promoted Eca109 cell migration and invasion *in vitro*

**Figure 3:**
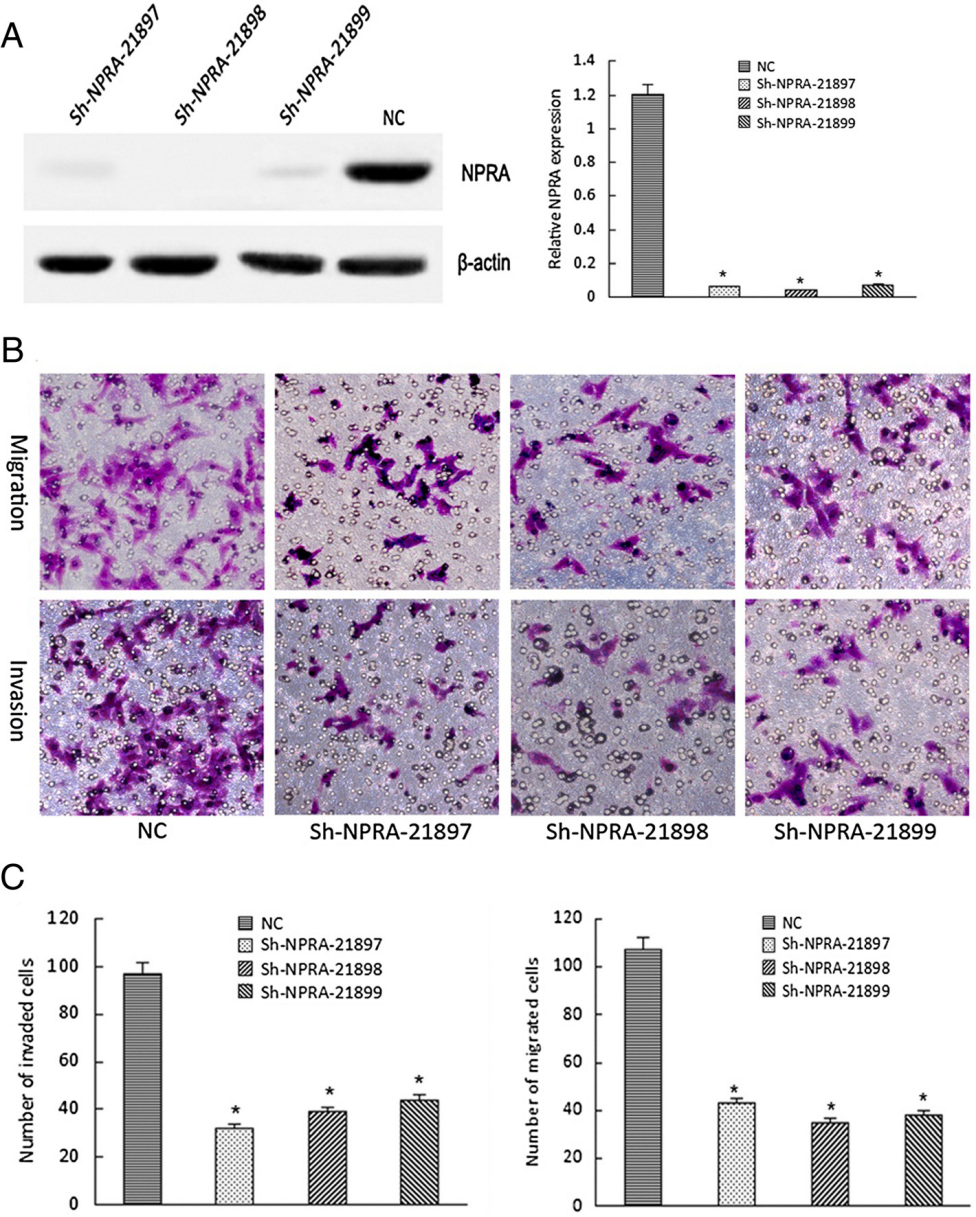
**Natriuretic peptide receptor-A (NPRA) promoted Eca109 cell migration and invasion *in vitro*. A**. western blot was used for evaluation of the silent effect of three Sh-RNA-NPRA; **B**. migration and invasion ability of Eca109 cells after transfection with Sh-RNA-NPRA by Transwell invasion and migration assays, **C**: Bar chart from **B** Data are presented as the mean ± SD; *P <0.05 compared with normal epithelial cell (NC) group using the Student’s *t*-test.

To evaluate the effects of NPRA on migration and invasion, a Matrigel invasion assay was used. Sh-RNA was used to suppress the expression of NPRA and western blot assay showed that the protein levels of NPRA were obviously decreased. Transwell migration assay showed that the migration ability of cells after transfection with sh-RNA-NPRA was obviously more reduced than in those transfected with sh-RNA-controls. Similarly, the ability of cells to invade that in downregulate NPRA expression groups was clearly lower than in control groups (*P* <0.05) (Figure 3).

### Blockage of natriuretic peptide receptor-A by sh-RNA suppressed the expression of MMP2 and MMP9

**Figure 4:**
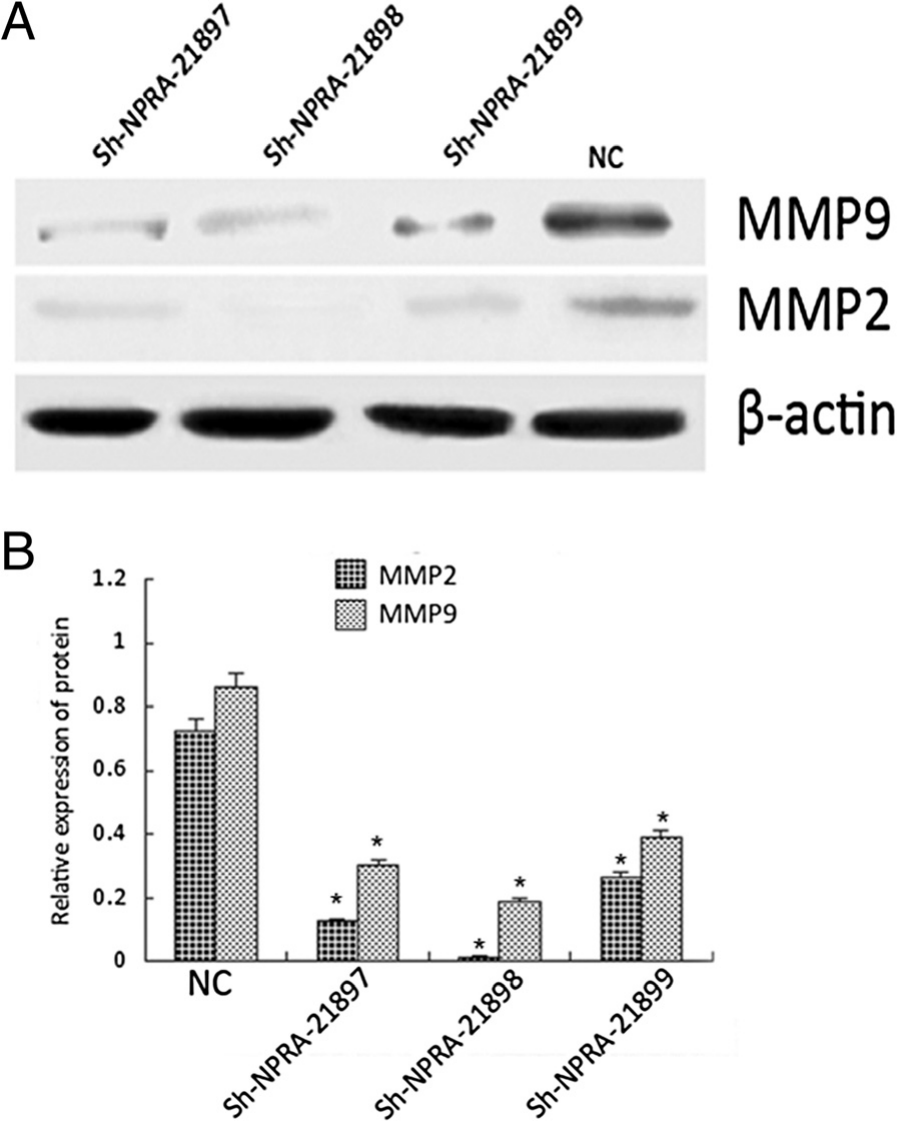
**Blockage of natriuretic peptide receptor-A (NPRA) by sh-RNA suppressed the expression of MMP2 and MMP9.** Sh-RNA against NPRA was used to downregulate the expression of NPRA and western blot was used to detect the expression of MMP2 and MMP9. **(A)**: western blot figure,** (B)**: Bar chart from **A**. *P <0.05 compared with normal epithelial cell (NC) group using the Student’s *t*-test.

To preliminarily investigate the mechanism of migration and invasion of NPRA in Eca109 cells, we used western blots to test the expression of MMP2 and MMP9 in Eca109 cells that were transfected with sh-RNA-NPRA. The results showed that the expression of MMP2, MMP9 and NPRA were all reduced (Figure 4).

## Discussion

In this study, we aimed to illustrate the expression level of NPRA in ESCC and the relationship of this expression with tumor invasion. This study demonstrates for the first time that NPRA is highly expressed in patients with ESCC, with a higher positive expression of NPRA correlating with TNM stage and histologic differentiation. Moreover, a significant finding of our study is the demonstration that downregulation of NPRA can suppress tumor invasion and migration and is associated with MMP2 and MMP9 expression. This will provide a direction for studying the specific mechanism of NPRA in malignancy metastasis.

Though immunohistochemistry and western blot, we found a distinctly differential expression and morphology in NPRA protein, mainly in the cytoplasm, between tumor cells and noncancer cells. This is similar to previous studies. Prior research indicates that NPRA is highly or lowly expressed on cells in many different tissues of various organ systems or cancers except for esophageal squamous carcinoma. Wang’s group found that NPRA is abundantly expressed in human prostate cancer (PCa) cells and tumorigenic mice, and that downregulation of NPRA can induce apoptosis in PCa cells; however, the expression of NPRA in nontumorigenic prostate epithelial cells is much lower than in PCa cells [16]. In experiments on NPRA downregulation, NPRA-deficient C57BL/6 mice showed significant anti-cancer activity [14], and NP73-120, which is the NH2-terminal peptide of ANP pro-hormone that can restrain the expression of NPRA, was used to investigate the effect in human lung cancer tumorigenesis. The results revealed that NPRA inhibited lung cancer metastasis and tumorigenesis [14]. Furthermore, in melanoma (B16), skin cancer, ovarian cancer (SKOV3 and ID8), gastric cancer, the expression of NPRA is also high [17,18]. In our experiment, we investigate the function of NPRA in human gastric cancer cells. We found that NPRA is abundantly expressed in human gastric cancer AGS cells, and downregulation of NPRA by sh-NPRA can induce apoptosis in AGS cells. In a word, our experimental data and the above evidence suggest that NPRA plays an important role in tumorigenesis. Recently, more and more poor prognostic markers have been reported in ESCC, such as Ep-CAM [19], Cyclin D1 [20], FADD [21], PTCH [22], hMLH1, hMSH2 [23] and so on [16]. Migration and invasion assays have demonstrated that the invasion and migration ability of Eca109 cells after transfection are significantly lower than for the control group. To investigate the mechanism of NPRA in invasion and migration, we detected MMP-2 and MMP-9 protein expression. The results showed that MMP-2 and MMP-9 were more obviously reduced in sh-RNA-NPRA compared with control group. Our data suggested that the NPRA-mediated invasion and migration role of Eca109 cells may involve MMP-2 and MMP-9 expression. Cancer invasion and metastasis is a complex and multistep process, which involves tumor cell adhesion, angiogenesis, matrix degradation and so on. The extracellular matrix (ECM) degradation plays an important role in the invasion and migration process [24-26]. Matrix metalloproteinases (MMPs) are the most important enzymes for degrading the ECM [27]. MMP-2 and MMP-9 are two family members that can specificity cleave type IV collagen and gelatin, which are the main structural components of the basal membrane, So, MMP2 is also called type IV collagenase [28]. Because the expression of MMP-2 and MMP-9 has been implicated in the development and progression of many tumors, such as prostate [29], colorectal [30], breast cancer [31] and cervical cancer [32], MMP2 has been deemed to be the most direct and important enzyme in invasion of cancer cells. Vellaichamy *et al*. [33] found that expression levels of MMP-2 and MMP-9 were increased three- to fivefold in cardiac fibrosis of mice lacking NPRA signaling; at the same time NF-kb signaling was enhanced fourfold. So they suggested that reduced NPRA signaling can activate MMP and is involved in NF-kb signaling activation, which leads to the development of cardiac hypertrophy, myocardial fibrosis, and congestive heart failure. However, our results showed the opposite for ESCC. In addition, Xiaoyuan Kong observed that NF-kb signaling activation and MMP-10 were reduced in the lung cancers of NPRA −/−mice [14], consistent with our results. So we hypothesize that NPRA mutates in some way in cancer, and this mutation may be involved in NF-kb signaling activation. In our future investigations, we will intensively study the differential regulation of NF-kb activity by the *NPRA* gene in human cancer.

## Conclusions

In conclusion, we demonstrate for the first time that NPRA was highly expressed in ESCC and associated with TNM stages, histologic differentiation and poor prognosis of ESCC. We also demonstrate that NPRA promotes Eca109 cell migration and invasion, which may regulate MMP-2 and MMP-9 activation. Nonetheless, there are plenty of shortcomings in our research, so further studies are needed to elucidate the specific molecular mechanisms of the NPRA/NF-kb/MMP2 and MMP9 pathways in ESCC. We believe that NPRA will be a new and effective target for use in diagnosing and treating ESCC.

## Abbreviations

ESCC: esophageal squamous cell carcinoma; MMP: matrix metalloproteinase; NPRA: natriuretic peptide receptor-A.

## Competing interests

The authors declare that they have no competing interests.

## Authors’ contributions

ZZ and YY carried out the molecular genetic studies, KS and ML participated in the specimen collection, HC and JZ arrange figures and tables, ZZ drafted the manuscript, JW and HL modify the manuscript. All authors read and approved the final manuscript.
